# Evaluation of specific humoral immune response and cross reactivity against *Mycobacterium tuberculosis* antigens induced in mice immunized with liposomes composed of total lipids extracted from *Mycobacterium smegmatis*

**DOI:** 10.1186/1471-2172-14-S1-S11

**Published:** 2013-02-25

**Authors:** María de los Angeles García, Reinier Borrero, Reynel Marrón, María E Lanio, Lien Canet, Oscar Otero, Ramlah Kadir, Siti Suraiya, Caridad Zayas, Yamilé López, Mohd Nor Norazmi, Maria E Sarmiento, Armando Acosta

**Affiliations:** 1 Molecular Biology Department Finlay Institute. Ave. 27 No. 19805, La Lisa. La Havana, Cuba. AP. 16017, CP11600; 2 Center for Protein Studies, Faculty of Biology, Havana University, Cuba; 3 School of Health Sciences, Universiti Sains Malaysia, Malaysia; 4 School of Medical Sciences, Universiti Sains Malaysia, Malaysia; 5 Institute for Research in Molecular Medicine, Universiti Sains Malaysia, Malaysia

## Abstract

The development of a new tuberculosis (TB) vaccine has become one of the main objectives of the scientific community. Protein antigens have been widely explored as subunit TB vaccines, however lipid antigens could be equally important to be used or included in such a vaccine. The aim of this study was to demonstrate the potential of a liposome formulation composed of an extract of lipids from *Mycobacterium smegmatis* (Ms) as a TB vaccine candidate. We evaluated the immunogenicity of this formulation as well as the cross reactive response against antigens from *Mycobacterium tuberculosis* (MTb) in BALB/c mice. We determined the anti-liposome IgG response in sera from TB patients and from healthy subjects who displayed a positive (PPD+) or negative (PPD-) tuberculin skin test. A significant increase in anti-liposome IgG (p<0.05) was detected in animals immunized with Bacille Calmette-Guérin (BCG) compared with all groups, and in the group immunized with liposomes from Ms (LMs) compared to animals immunized with either LMs adjuvanted with aluminium (LMs-A) or the negative control group (phosphate buffered saline, PBS) respectively. With respect to the cross reactive response against a cocktail of cell wall antigens (CWA) from MTb, significantly higher IgG levels were observed in animals immunized with BCG and LMs compared to negative controls and either, aluminium-adjuvanted liposomes (LMs-A) or montanide (LMs-M) (p<0.05). Furthermore, the anti-liposome IgG response was significantly superior in sera from pulmonary TB patients compared to PPD+ and PPD- healthy subjects (p<0.001) suggesting the expression of these antigens *in vivo* during active MTb infection. The results obtained provide some evidence for the potential use of liposomes containing total lipid extracts of Ms as a TB vaccine candidate.

## Background

TB remains one of the infectious diseases that cause great morbidity and mortality worldwide. BCG, the current vaccine against TB offers poor or no protection against the most common clinical form of the disease, pulmonary TB in adults. It has shown variability in efficacy ranging from 0% to 80 % in several clinical trials around the world [1]. Worst still, the epidemiological situation of this disease is worsening in many parts of the world. Therefore, there is an urgent need for a new or improved TB vaccine.

It has previously been shown that liposome formulations composed of total lipid extracts from Ms possess immune-adjuvant properties [2,3]. It is therefore important to study the potential of such formulations as immunoprophylactic tools against TB, considering the fact that there is a high level of antigenic and genomic homology between Ms and MTb [4] and that there are great similarities in the lipid components of their cell walls.They have similar mechanisms of cell wall synthesis [5] and glycopeptidolipids of Ms recognize the same receptors as those of MTb [6].

This paper is an initial attempt to demonstrate the potential of a liposome formulation composed of total lipid extract from Ms as a possible TB vaccine candidate.

An evaluation of the anti-LMs specific IgG response and the cross-reactive IgG response against CWA from MTb in BALB/c mice immunized with LMs was carried out. Additionally, the anti-LMs specific IgG response in sera of TB patients as well as that of PPD+ and PPD- healthy subjects was determined.

## Materials and methods

Ms mc^2^155 strain [7] (from the collection of the National Reference Laboratory of Tuberculosis, Pedro Kouri Institute, Cuba) was used. Cultures were grown in 1% (w/v) yeast extract (Merk, Germany), 0.5% (v/v) glycerol (Riedel de Haen, Germany), 0.4% (v/v) Tween 80 (Fluka, Germany), in 8% nutrient broth (Biocen, Cuba) for 48h with agitation (200 rpm) at 37ºC. The purity of the culture was evaluated by Ziehl-Neelsen staining [8].

Total lipid extraction from Ms was accomplished using the technique described by Bligh and Dyer [9] and the liposomes containing these lipids were obtained by the dehydration-rehydration method [10]. The size of the vesicles was determined by transmission electron microscopy (TEM) and dynamic light scattering using a Brookhaven *ZetaPlus* (Brookhaven Instruments Worcs, UK).

Fifty female BALB/c mice (6-8 weeks) were used in the experiments. The procedure was carried out according to the international regulations for laboratory animal experimentation [11]. Five groups of animals (n=10) were inoculated subcutaneously with either PBS; BCG, (Moreau strain, EPB ”Carlos J. Finlay”, Cuba) (10^6^ CFU); LMs composed of 1mg of total lipids from Ms; LMs-A (LMs 1mg + alum Alhydrogel, Sigma, 1 mg) or LMs-M (LMs 1mg + Montanide, Seppicc, France). Two doses at 0 and 21 days were administered. The group immunized with BCG only received one dose on day 0. Blood samples were collected 42 days after the first immunization. The blood was centrifuged and the sera stored at -20ºC until use.

Sera from TB patients, PPD+ and PPD- healthy subjects were collected from the Universiti Sains Malaysia Hospital (HUSM). The protocols of the study were conducted according to the ethical guidelines as approved by the Human Ethics Committee of USM and written informed consent from participants was obtained.

An indirect ELISA was performed to measure the anti-LMs IgG response. Briefly, the plates were coated with 10µg/mL of the LMs formulation for 16h at 4°C and sera from mice were diluted at 1:50 and incubated for 1h at 37°C. A HRP conjugated rabbit anti mouse IgG (Sigma) at a dilution of 1:3000 was used and incubated for 1h at 37°C. The reaction was developed with a solution (100 µl /well) of *o*-phenylendiamine (OPD; Sigma) (5 mg in 12.5 ml 0.1 M sodium citrate buffer, pH 5 + 5 µl 30% (v/v) H_2_O_2_). After 20 to 30 min the reaction was stopped with 2N H_2_SO_4_ (50µl/well) and the absorbance was read at 492nm. To evaluate the cross-reactivity against the cell wall antigens (CWA) of MTb, a cocktail of these antigens was used to coat the ELISA plate at a concentration of 10µg/mL for 16h at 4°C.

A similar procedure was also followed to measure the anti-LMs IgG response in sera from TB patients, PPD+ and PPD- healthy subjects except that the secondary antibody used was a HRP conjugated rabbit anti human IgG (Sigma) at 1:6000 dilution.

## Results and discussions

Using the described method, liposomes of between 50 to 200 nm were obtained (data not shown). The composition of the liposomes were deduced to consist of phosphatidylethanolamine, phosphatidylglycerol, cardiolipin, phosphatidylinositol and phosphatidylinositol mannosides, based on a similar methodology used by Faisal et al [12].

**Figure 1:**
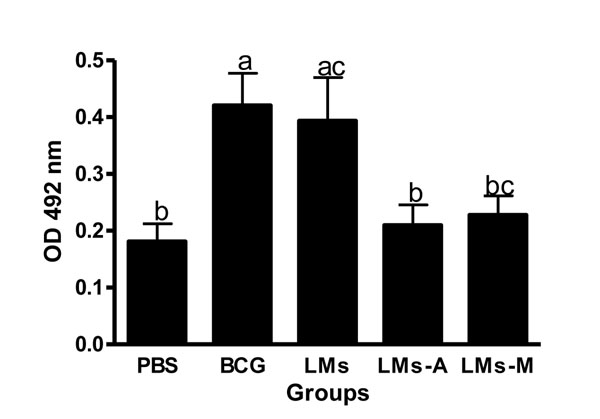
**Humoral immunity induced by LMs.** Total IgG response against LMs of BALB/c mice (n=10/group) immunized with either PBS, BCG, LMs, LMs-A and LMs-M. Results are presented as mean ± SD. One way ANOVA and Tukey multiple comparison tests were used to analyse the data. Different letters means statistical difference between groups (p<0.05).

**Figure 2:**
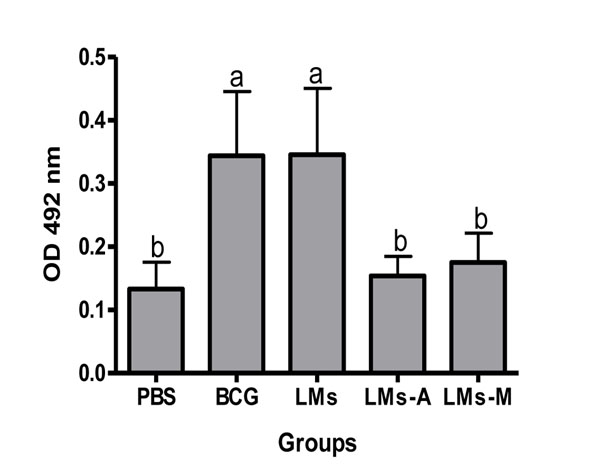
**Cross reactivity against CWA from MTb.** Total IgG response against CWA from MTb in BALB/c mice (n=10/group) immunized with either PBS, BCG, LMs, LMs-A, LMs-M. Results are presented as mean ± SD. One way ANOVA and Tukey multiple comparison tests were used to analyse the data. Different letters means statistical difference between groups (p<0.05).

In this report we demonstrated that liposomes composed of total lipids of Ms induced specific IgG response against LMs antigens in BALB/c mice (p<0.05). BCG immunization induced a significant IgG response compared to all other groups, except to LMs. Interestingly, the LMs inmunized group developed a higher IgG response compared to the negative control group and animals immunized with LMs-A (p<0.05 ) (Fig. 1). Based on the results obtained in the group immunized with BCG, it seems that BCG lipids and those of Ms share similar epitopes. Regarding the cross-reactive response against CWA from MTb in BALB/c mice, it was observed that significantly higher IgG levels was obtained from the BCG- as well as LMs-immunized groups compared to the negative control group and groups immunized with LMs-A and LMs-M respectively (p<0.05) (Fig. 2). This could be probably due to the masking effect or the structural disruption with a possible epitope modification caused by the adjuvants themselves to the lipidic components. In contrast to what was found by Rodriguez et al. [13], where the activity of Ms derived proteoliposomes was enhanced by the adjuvant, the LMs without adjuvants gave the better IgG response. These results could be a consequence of different structural stability of proteoliposomes or LMs respectively in presence of adjuvants.

The cross-reactive response observed in the group immunized with LMs suggests that there are great structural antigenic similarities between MTb and Ms.

**Figure 3:**
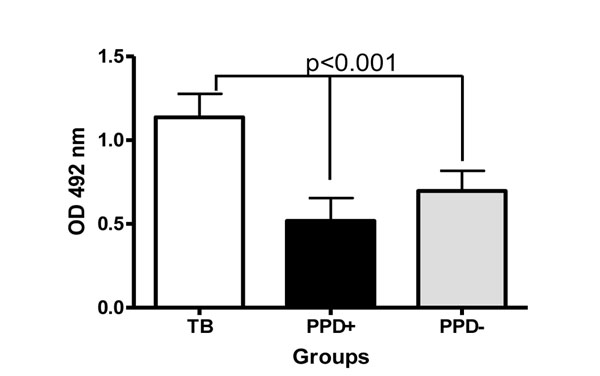
**Anti-LMs IgG reactivity of sera from TB patients, PPD+ and PPD- subjects.** Results are presented as mean ±SD. One way ANOVA and Tukey multiple comparison tests were used to analyse the data.

Interestingly, as shown in Fig. 3, the anti-LMs IgG response was significantly higher in TB patients than in PPD- and PPD+ healthy subjects (p<0.001). One possible explanation for this result is that during active TB infection, epitopes similar to those contained in the liposomes derived from Ms are expressed by MTb.

Immunization with LMs could be a promising strategy to elicit immune responses against antigens presented to the immune system during MTb infection *in vivo*.

## Conclusions

This is the first report studying the potential of liposomes composed of total lipids of Ms as a possible TB vaccine candidate. Here the vector of the vaccine is also a relevant antigenic structure demonstrated by the capability of this formulation to induce specific IgG response and cross-reactivity against MTb in BALB/c mice. In addition, components of the liposome formulation were recognized by sera from TB patients suggesting the possible presence of these epitopes in MTb during active TB. These results support the future evaluation of LMs as an experimental vaccine candidate against TB.

## Competing interests

The authors declare that they have no competing financial interests.

## Authors' contributions

All author have read and approved the final manuscript. MAG, RB and RM participated in the production of liposomes, animal experiments, evaluation of immunogenicity and cross reactivity, data analyses and writing of the manuscript. MEL and LC participated in production of liposomes. SS, CZ, RK and OO participated in the evaluation of immunogenicity and cross reactivity. YL participated in writing of the manuscript. MNN, MES, AA conceived the study, experimental design, participated in data analyses and in writing and finalizing of the manuscript.

## Declarations

This article has been published as part of *BMC Immunology* Volume 14 Supplement 1, 2013: Proceedings from Delivery Systems and Current strategies to drug design. The full contents of the supplement are available online at http://www.biomedcentral.com/bmcimmunol/supplements/14/S1.
